# Intravenous ferric carboxymaltose for the management of iron deficiency and iron deficiency anaemia in children and adolescents: a review

**DOI:** 10.1007/s00431-022-04582-w

**Published:** 2022-09-02

**Authors:** Aysegül Aksan, Fred Zepp, Sangeetha Anand, Jürgen Stein

**Affiliations:** 1 Interdisciplinary Crohn Colitis Centre Rhein-Main, Frankfurt, Germany; 2 Institute of Nutritional Science, Justus-Liebig University, Giessen, Germany; 3 University Medical Center of the Johannes-Gutenberg University, Mainz, Germany; 4 Vifor Pharma, Opfikon, Switzerland; 5 Department of Gastroenterology and Clinical Nutrition, DGD Clinics Sachsenhausen, Frankfurt, Germany

**Keywords:** Iron deficiency, Ferric carboxymaltose, Children, Adolescents, Paediatrics

## Abstract

Iron deficiency is the primary cause of anaemia worldwide and is particularly common among children and adolescents. Intravenous (IV) iron therapy is recommended for paediatric patients with certain comorbidities or if oral iron treatment has been unsuccessful. IV ferric carboxymaltose (FCM) has recently been approved by the US Food and Drug Administration for use in children aged > 1 year. This narrative review provides an overview of the available publications on the efficacy and safety of IV FCM in children and adolescents. A literature search using PubMed and Embase yielded 153 publications; 33 contained clinical data or reports on clinical experience relating to IV FCM in subjects < 18 years of age and were included in the review. No prospective, randomised controlled studies on the topic were found. Most publications were retrospective studies or case reports and included patients with various underlying conditions or patients with inflammatory bowel disease. Efficacy data were included in 27/33 publications and improvements in anaemia, and/or iron status parameters were reported in 26 of them. Safety data were included in 25/33 publications and were in line with the adverse events described in the prescribing information.

*Conclusion:* The available publications indicate that IV FCM, a nanomedicine with a unique and distinctive therapeutic profile, is an effective and generally well-tolerated treatment for iron deficiency or iron deficiency anaemia in children and adolescents. Despite the wealth of retrospective evidence, prospective, randomised controlled trials in the paediatric setting are still necessary.

**Table:** 

**What is Known:** *• Iron deficiency and iron deficiency anaemia are usually managed using oral iron therapy, but intravenous iron therapy is recommended for certain paediatric patients.* *• Intravenous ferric carboxymaltose (FCM) has recently been approved in the US for use in children aged > 1 year.*	
**What is New:** *• Despite evidence that FCM is effective and generally well tolerated in children and adolescents, so far, only retrospective studies, non-randomised uncontrolled prospective studies, or case reports have been published in full.* *• There is a strong need for prospective, randomised controlled trials on FCM in the paediatric setting.*

**What is Known:**

*• Iron deficiency and iron deficiency anaemia are usually managed using oral iron therapy, but intravenous iron therapy is recommended for certain paediatric patients.*

*• Intravenous ferric carboxymaltose (FCM) has recently been approved in the US for use in children aged > 1 year.*

**What is New:**

*• Despite evidence that FCM is effective and generally well tolerated in children and adolescents, so far, only retrospective studies, non-randomised uncontrolled prospective studies, or case reports have been published in full.*

*• There is a strong need for prospective, randomised controlled trials on FCM in the paediatric setting.*

## Introduction

Iron deficiency (ID) is the most common cause of anaemia worldwide [1] and particularly affects children and adolescents as well as pre-menopausal women, pregnant women and the elderly [2–4]. In 2019, ID was the leading risk factor for attributable disability-adjusted life years for the 10–24 years age group [5]. The reported prevalence of ID and iron deficiency anaemia (IDA) in children varies widely. A review of studies across Europe found that ID prevalence in young children varied depending on socioeconomic status and type of milk consumed (i.e. formula, human or cow’s milk) [6]. Prevalence of ID in pre-school-aged children ranged from 3 to 48%, while the prevalence of IDA was < 5% in Northern and Western Europe and 9–50% in Eastern Europe [6]. In the USA, the prevalence of ID and IDA in pre-school-aged children was estimated to be 7.1% and 1.1%, respectively [7]. Among adolescents, the prevalence of IDA may be as high as 25–30% in low–middle social development index countries [8].

The first-line treatment for ID/IDA is generally correction of the iron deficiency with iron-rich foods and/or oral iron supplementation [4, 9]. Various oral iron preparations are available, but ferrous sulphate is the most commonly used worldwide [4]. Orally administered iron (liquid or tablet formulations) is generally effective, but side effects, as well as difficulty swallowing tablets and poor taste, can lower adherence to therapy, especially in children [4, 10]. Intravenous (IV) iron therapy provides an alternative option that can be considered as a second-line treatment when oral iron therapy has been unsuccessful [4, 9]. IV iron therapy can also be used as an appropriate first-line treatment for specific patient groups, including children with gastrointestinal disorders, chronic kidney disease (CKD) or restless legs syndrome, and children on long-term parenteral nutrition [9, 11–16].

IV iron preparations have been available for some time, and iron sucrose is a widely used IV iron for the treatment of ID/IDA. However, iron sucrose requires repeated dosing over alternate days [4]. More recently developed IV iron preparations, such as ferric carboxymaltose (FCM), ferumoxytol and iron isomaltoside 1000, are optimised for dosing and allow correction of ID with a single infusion [4]. FCM can be administered as a single dose in 15 min [17] and has recently been approved by the US Food and Drug Administration (FDA) for the treatment of IDA in paediatric patients aged > 1 year who have either intolerance or an unsatisfactory response to oral iron [18]. In Europe, FCM is currently approved only for patients ≥ 14 years of age [17].

FCM belongs to a group of pharmaceutical compounds known as non-biological complex drugs (NBCDs). NBCDs are typically composed of large high-molecular weight molecules and, often, nanoparticular structures [19]. For nanomedicines such as FCM, a strictly regulated manufacturing process is fundamental to the therapeutic properties of the final medicinal product. Given the complexity in the characterisation of these nanomedicines, even minor changes in production, storage and handling can influence the safety and effectiveness of the final product [20]. Therefore, derived products or similar iron products cannot be assumed to be equivalent to FCM without clinical evidence [19].

This narrative review aims to summarise the available clinical evidence on FCM in the paediatric setting and to identify key data and knowledge gaps. We conducted a literature search to identify publications on the efficacy and safety of FCM in children and adolescents (including those aged < 14 years) and have presented our findings here.

## Methods

A literature search was conducted on 16 February 2021 using PubMed and Embase. The search terms used were as follows: (ferric carboxymaltose OR Ferinject) AND (children OR paediatric OR infant OR child OR neonate OR newborn OR adolescent OR juvenile).

Search results were screened to remove duplicates, then the remaining publications were reviewed based on the abstracts to identify English language articles of potential relevance. Full-text articles were obtained for all potentially relevant publications and selected for inclusion in the narrative review if they included novel clinical data or clinical experience on the use of FCM in patients aged < 18 years. Publications without novel clinical evidence (e.g. reviews, editorials and guidelines) were excluded. Congress abstracts were excluded if the data were subsequently available in a full publication. Publications were excluded if they were related to the use of FCM in adults aged ≥ 18 years.

Efficacy and safety findings for FCM were reviewed and summarised. Outcomes considered as efficacy findings included (but were not limited to) change in iron status laboratory parameters from pre- to post-treatment, percentage of patients achieving target values for iron status parameters, resolution of anaemia and change in iron status parameters compared with the control group. Outcomes considered as safety findings included any treatment-emergent or treatment-related adverse events and incidence of hypophosphataemia.

## Results

**Fig. 1:**
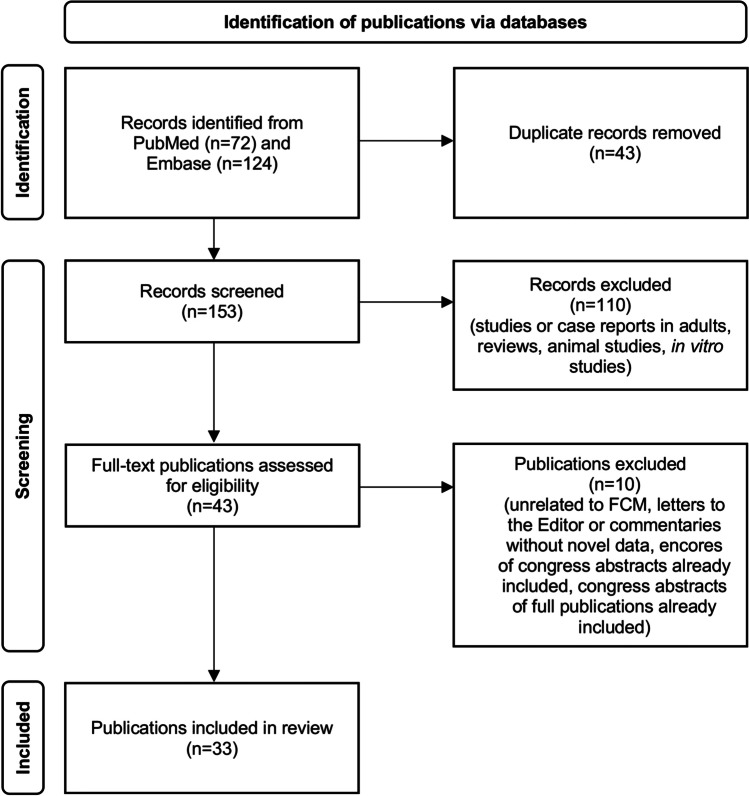
PRISMA flow diagram summarising the literature search steps for the identification of publications on the efficacy and safety of FCM in children and adolescents aged < 18 years

The literature search yielded 153 unique publications (Fig. 1). Of these, 33 were related to the use of FCM in children or adolescents < 18 years and were included in this narrative review (Table 1). The 33 publications evaluated in this analysis consisted of 19 retrospective studies [21–39], two prospective studies [40, 41], eight case reports [42–49], one case series (included three case reports [50]), one audit [51], one pharmacokinetic/pharmacodynamic modelling study [52] and one letter to the editor [53].

**Table 1:** Summary of publications on FCM in children or adolescents aged < 18 years

**Publication**	**Study population**	**Age (number of subjects)**	**Main efficacy findings**	**Main safety findings**
**Retrospective studies**				
Athiana et al. [21]	Intestinal failure	< 2 years (*N* = 14)	Complete or partial normalisation of Hb, ferritin and MCV after 1–3 months	No AEs reported
Cococcioni et al. [22]	IBD	3–18 years, mean 12.5 years (*N* = 128)	Significant improvements in Hb (106.36 to 122.73 g/L, *p* < 0.001), ferritin (67.29 to 218.15 μg/L, *p* < 0.001), iron (6.060 to 11.737 μmol/L, *p* < 0.001), MCV (74.424 to 80.602 fL, *p* < 0.001) and ESR (32.92 to 25.99 mm/h, *p* < 0.05) after 4–6 weeks	Of 25 patients with low serum phosphate, two had severe hypophosphataemia, one patient had anaphylactic reaction and two patients had pruritus and fever; no AEs reported for patients < 6 years
Crighton et al. [23]	IDA (various aetiologies)	< 14 years, median 9.3 years (*N* = 60)	Significant improvements in Hb and MCV (*p* < 0.001)	Three infusions associated with mild AEs and one episode of extravasation
Dargan et al. [24]	IDA (various aetiologies)	Not reported (paediatric hospital setting) (*N* = 120*)	Preliminary data demonstrate an increase in Hb after treatment with FCM	Not reported
DelRosso et al. [25]	Restless legs syndrome	Mean 11.5 years for the FCM group (*N* = 52, 28 received FCM)	Significant improvements in ferritin (13.9 to 112.9 μg/L, *p* < 0.000001), TSAT (22.8 to 31.7%, *p* < 0.0001) and TIBC (366.7 to 302.0 μg/dL, *p* < 0.0000035) after 8 weeks; ferritin values higher with FCM vs oral iron (*p* < 0.000001); restless legs syndrome was reported to be resolved or improved in all children treated with FCM (vs 62.5% with oral iron)	AEs reported in 17.8% of patients treated with FCM and included light-headedness and GI discomfort
DelRosso et al. [26]	Restless sleep disorder	5–18 years, median 13 years for FCM group (*N* = 30, 15 received FCM)	Significantly higher median ferritin (124.0 vs 34.0 μg/L, *p* < 0.00003), iron (103.0 vs 77.0 μg/dL, *p* < 0.0084) and transferrin (31.0 vs 22.5 mg/dL, *p* < 0.004) and lower median TIBC (298.0 vs 333.0 μg/dL, *p* < 0.02) after 8 weeks; restless sleep disorder symptom improvement was more pronounced with FCM vs oral iron (*p* < 0.023)	One patient in the FCM group had syncope
Hachemi et al. [27]	IBD	7–19 years, median 16 years (*N* = 56, complete data for 40)	Significant increases in median Hb (19 g/L), ferritin (117 mg/L), iron (8.5 μg/L), MCV (7.5 fL) and Ht (0.04 L/L) after 4–6 weeks	2/40 patients developed allergic reactions with fever, shivering and vomiting
Hong et al. [28]	IDA (various aetiologies)	< 14 years (*N* = 176)	Improvements in Hb (106 to 122.3 g/L), iron (6.7 to 11.7 μmol/L) and ferritin (20.2 to 85.3 μg/L)	One patient had hypotension, four had a rash and two had a fever
Jacobson-Kelly et al. [29]	IBD	< 21 years, median 15.4 years (*N* = 8007, 448 received IV iron*)	Not reported	Not reported
Kirk et al. [30]	IDA (various aetiologies)	2 months to 20.3 years, median 9.2 years (*N* = 225)	Significant improvements in mean Hb (9.4 to 11.7 g/dL, *p* < 0.001), ferritin (33.4 to 108.2 ng/dL, *p* < 0.001) and MCV (76.4 to 81.9 fL, *p* < 0.001) after 4–12 weeks	Hypophosphataemia occurred after 44/313 (14%) infusions, in 40 patients
Knafelz et al. [31]	IBD	3–17 years, median 12 years (*N* = 56)	Improvements in median Hb (104 to 124 g/L), Ht (0.32 to 0.36 L/L), MCV (73.3 to 78.3 fL) and iron (6.7 to 13.4 μg/L) after 6 weeks	One patient developed shivering and fever
Laass et al. [32]	GI disorders	0–18 years, mean 11.8 years and 72 patients	Improvements in mean Hb (9.5 to 11.9 g/dL), MCV, ferritin and TSAT over 12 weeks	Two patients had mild urticaria, and one had mild oedema
Ozsahin et al. [33]	ID/IDA (various aetiologies)	18 months–18 years (19% < 6 years, 22% ≥ 6 and < 12 years, 59% ≥ 12 and < 18 years) (*N* = 144)	Of patients with complete data, 85% achieved the target ferritin level (≥ 30 μg/L) after 6–12 weeks, 83% of patients with IDA showed a complete or partial haematological response (defined as target reached for Hb, ferritin, MCV and MCH, or increment of ≥ 10 g/L in Hb from baseline at 6–12 weeks post-treatment)	11 patients had AEs in the clinic (six had tiredness and faintness, probably due to premedication with antihistamine; one was lightly agitated due to the procedure; four had immediate events possibly associated with FCM: urticaria, nausea, headache and discomfort), and five patients reported potentially related AEs during the 96-h follow-up (one had fever, nausea and diarrhoea; one had pain in the legs and long bones, abdominal pain, fever and nausea; one had abdominal pain, pain in the long bones and headache; one had pain in the legs and long bones and abdominal pain; one had asthenia, headache, abdominal pain and pain in the legs)
Papadopoulos et al. [34]	IBD	3–17 years, median 14 years (all patients who received FCM were ≥ 12 years) (*N* = 41, 35 received IV FCM)	Efficacy by treatment group not reported	2/35 patients who received FCM developed a mild rash
Posod et al. [35]	IDA (various aetiologies)	Median 12.7 years (*N* = 36)	Efficacy not reported	Hypophosphataemia occurred in 8/71 FCM infusions. Potential gender effect, with girls more likely to have a decrease in plasma phosphate
Powers et al. [36]	IDA (various aetiologies)	9 months to 18 years, median 13.7 years (*N* = 72)	Of the 53 patients with follow-up tests, 52 (98%) had a complete or partial haematological response (defined as normalisation of Hb and MCV measurements and ferritin ≥ 15 ng/mL or increment of ≥ 1 g/dL in Hb above preinfusion level)	Seven patients reported minor transient AEs: one had dyspnoea, four had pruritus or urticaria, one had tingling and one had extravasation
Sasankan et al. [37]	Gastroenterology patients	< 18 years, median 14 years (42% were < 14 years, 11.5% were < 5 years) (*N* = 61)	Significant improvements in median Hb (108 to 126 g/L, *p* < 0.00001) and MCV (80 to 84 fL, *p* = 0.0007) after 1 month; anaemia corrected in 94% of children	One patient had skin staining, and one patient had tingling and bruising
Spinner et al. [38]	Systolic heart failure	≤ 18 years, median 8.1 years (*N* = 42)	Significant improvements in median iron (38 to 67 μg/dL, *p* < 0.001), ferritin (38 to 142 ng/mL, *p* < 0.001), transferrin (293 to 261 mg/dL, *p* = 0.016) and TSAT (9.0 to 18.0%, *p* < 0.001) for the 25 patients with follow-up tests within 12 weeks	AEs occurred after 4/55 infusions (two fever, two nausea); one patient with a recent cardiac arrest died of recurrent arrest 24 h after infusion
Tan et al. [39]	IDA (various aetiologies)	1–13 years, median age 10.7 years (*N* = 51)	Improvements in median Hb (8.9 to 12.2 g/dL), iron (3.0 to 10.6 μmol/L) and TSAT (4.5 to 17%) after a mean of 2.4 months; one patient with inflammatory enteritis and one with very early onset IBD did not show improvements in the measured parameters likely due to their underlying disease	No AEs reported
**Prospective studies**				
Carman et al. [40]	IBD	6–18 years, median 14 years (*N* = 101)	Patients with IDA: improvements in median Hb (111 to 132 g/L, *p* < 0.001) and TSAT (8 to 20%, *p* < 0.001), and 64% had resolution of anaemia after a median of 8 weeks; patients with ID without anaemia: improvements in median TSAT (11 to 20%) and 81% had resolution of ID after a median of 8 weeks	Two patients had itch, urticarial rash and low-grade fever
Valério de Azevedo et al. [41]	CD	6–18 years, median 15.5 years (all patients who received FCM were ≥ 14 years) (*N* = 19, 10 received FCM)	Improvements in median Hb (10.4 to 13.1 g/dL) after 4–6 weeks	One patient had minor headaches, and one patient had a fever
**Case reports/series**				
Abdelmahmuod and Yassin [42]	Lymphocytopenia	17 years (*N* = 1)	Anaemia and lymphocytopenia improved after therapy; Hb increased from 5.2 to 11.0 g/dL	Not reported
Beverina et al. [43]	Extreme IDA	13 years (*N* = 1)	Hb increased from 33 to 79 g/L after 12 days and to 144 g/L after about 7 months	No AEs reported
Daignault et al. [44]	Burkitt’s lymphoma	16 years (*N* = 1)	Patient was initially treated with red blood cell transfusion and oral ferrous sulphate, then received FCM 2 weeks later due to continued anaemia. However, she continued to have symptomatic anaemia and was eventually diagnosed with Burkitt’s lymphoma	Not reported
Harris et al. [45]	CD	17 years (*N* = 1)	Not reported	Two days post-infusion, a patchy, brown discolouration of the skin surrounding the initial cannula site was reported. This case represents one of three occurrences of skin staining secondary to iron extravasation recognised within the department between December 2014 and August 2016
Harris et al. [50]	IBD	16–17 years (*N* = 3)	Case 1: Hb increased from 109 to 111–127 g/L after 1 month; case 2: Hb increased from 115 to 121–132 g/L after 8 weeks; case 3: iron 8 μmol/L and TSAT 13% pre-FCM and remained low 6 weeks after the second infusion (iron 5 μmol/L and TSAT 11%)	All three patients experienced hypophosphataemia
Hönemann et al. [46]	Post-traumatic anaemia patient who refused blood transfusion	17 years (*N* = 1)	Improvement in Hb concentration (4.2 to 11.1 g/dL after 17 days)	No AEs reported
Joseph et al. [47]	IRIDA and at risk of hypersensitivity reactions	Adolescent (age not given) (*N* = 1)	A 12-step FCM desensitisation protocol resulted in tolerated FCM and corrected anaemia	Not reported
Pérez-Ferrer et al. [48]	Cardiac surgery and factor VII deficiency, parents did not consent to blood transfusion	5 years (*N* = 1)	Improvement in Hb (12.5 to 14.5 g/dL) and Ht (36.6 to 47.1%) within 12 days of erythropoietin and FCM treatment; no transfusion of blood products required	Not reported
Shrinkhal et al. [49]	Anaemic retinopathy, megaloblastic anaemia with thrombocytopenia	16 years (*N* = 1)	Changes in iron status not reported; retinal haemorrhage spontaneously resolved with clearance of fovea and the patient gained vision	Not reported
**Others**				
Crook et al. [51]	IBD	4–17 years, mean 12 years (*N* = 29)	Improvements in Hb (87 to 123 g/L for overall population; 83 to 115 g/L for 4–11-year-olds) and ferritin (7.5 to 137 μg/L for overall population; 6.9 to 137 μg/L for 4–11-year-olds) after 6–10 weeks; 76% had Hb level recover to within normal range	No AEs reported
Jones et al. [52]	IDA (aetiology not reported)	1.5–17.5 years and 33 patients	Not reported	Not reported
Mantadakis and Roganovic [53]	IDA (various aetiologies)	8–17.9 years, median 12 years (*N* = 15)	Improvements in median Hb (73 to 126 g/L) at > 4 weeks	Painless extravasation in one patient that led to mild iron staining of the forearm

*AE* adverse event, *CD* Crohn’s disease, *ESR* erythrocyte sedimentation rate, *FCM* ferric carboxymaltose, *GI* gastrointestinal, *Ht* haematocrit, *Hb* haemoglobin, *IBD* inflammatory bowel disease, *ID* iron deficiency, *IDA* iron deficiency anaemia, *IRIDA* iron-refractory iron deficiency anaemia, *IV* intravenous, *MCH* mean cell haemoglobin, *MCV* mean corpuscular volume, *TIBC* total iron binding capacity, *TSAT* transferrin saturation

^*^Some patients received FCM, but the number of patients was not reported

The patient groups included 10 studies on children with ID/IDA associated with different underlying conditions [23, 24, 28, 30, 33, 35, 36, 39, 52, 53]; in three of these, all the children were under 14 years of age [23, 28, 39]. There were also eight studies (including the audit and two prospective studies) on children with inflammatory bowel disease (IBD) [22, 27, 29, 31, 34, 40, 41, 51] and two studies on children with varying gastrointestinal disorders [32, 37]. Other patient groups included in the studies were restless legs syndrome (one study [25]), restless sleep disorder (one study [26]), heart failure (one study [38]) and intestinal failure (one study in children under 2 years of age [21]). Furthermore, there were case reports of adolescents with IBD (three cases [50]), children unable to receive blood transfusions (one case of a 5-year-old undergoing cardiac surgery [48] and one case of a 17-year-old with post-traumatic anaemia following a road accident [46]), extreme IDA (one case in a 13-year-old [43]), Crohn’s disease (one case of a 17-year-old [45]), anaemic retinopathy (one case of a 16-year-old [49]), lymphocytopenia (one case of a 17-year-old [42]), Burkitt’s lymphoma (one case of a 16-year-old [44]) and iron-refractory iron deficiency anaemia (IRIDA) with prior anaphylaxis to IV iron (one case of an adolescent whose age was not reported [47]).

### Efficacy of FCM in children and adolescents

Findings on the efficacy of FCM in children and/or adolescents were reported in 27 of the 33 publications (Table 1) [21–28, 30–33, 36–44, 46–48, 50, 51, 53]. In 26 of the 27 publications that included efficacy results, FCM treatment (in most cases a single dose) was associated with improvement in anaemia and/or different iron status parameters, including improvements in levels of haemoglobin (22 publications), ferritin (12 publications), mean corpuscular volume (10 publications), iron (six publications) and transferrin saturation (five publications) (Table 1). Only one of the publications that included efficacy results (a case report) reported no improvement following FCM treatment (anaemia persisted), and the patient was eventually diagnosed with Burkitt’s lymphoma [44].

The efficacy of FCM in the < 14 years age group has been investigated in three single-centre retrospective studies in children with IDA associated with different underlying conditions [23, 28, 39]. In the largest of these studies, involving 176 children, FCM treatment was associated with improvements in haemoglobin, iron and ferritin levels [28]. In another study in 60 children, there were significant improvements in haemoglobin and mean corpuscular volume following FCM treatment, although the change in ferritin levels did not reach statistical significance [23]. In addition, a study of 51 children reported improvements in haemoglobin, iron and TSAT following FCM treatment [39]. The use of FCM has also been reviewed retrospectively in children < 2 years old with intestinal failure and ID [21]. All 14 children who received one or two doses of FCM responded with complete or partial normalisation of markers for ID.

The largest study of FCM in the paediatric age range was a single-centre retrospective study that included 225 patients aged 2 months–20.3 years with IDA of various aetiologies [30]. While the primary objective of this study was to assess phosphate levels in children treated with FCM, iron parameters were also recorded, showing significant improvements in haemoglobin, mean corpuscular volume and ferritin values [30]. Another large study to report the efficacy of FCM in patients up to 18 years old was a single-centre retrospective study that included 144 patients with ID/IDA due to various causes and poor response to oral iron, receiving a single dose of FCM [33]. Of the 117 patients with complete data, 85% achieved the target ferritin level of ≥ 30 µg/L; of the 82 patients with IDA and complete data, 83% achieved a complete or partial haematological response. Other large studies were a retrospective study in two centres involving 128 patients aged 3–18 years with IBD and IDA [22] and a single-centre prospective study including 101 patients aged 6–18 years with IBD and ID/IDA [40]. In both studies, iron status parameters improved following FCM treatment (most patients received one dose). In the prospective study, 81% of patients with ID without anaemia showed resolution of ID after IV FCM treatment [40].

Only three small studies compared FCM with another therapy. In a prospective study of 19 children aged 6–18 years with Crohn’s disease and IDA, 10 children (all aged ≥ 14 years) received FCM and nine received iron sucrose [41]. The two therapies were not directly compared but both groups showed similar improvements in median haemoglobin levels (10.4 to 13.1 g/dL with FCM, 10.6 to 12.3 g/dL with iron sucrose). In a retrospective case series, 28 children (mean age 11.5 years) with restless legs syndrome and ferritin levels < 50 µg/L were treated with a single dose of FCM and compared with 24 controls (age- and sex-matched children with restless legs syndrome treated with oral iron) [25]. Ferritin levels were significantly higher in the FCM group 8 weeks after the infusion compared with the control group, and restless legs syndrome had resolved or improved in all children treated with FCM (vs 62.5% of controls). Finally, in another retrospective study in which children aged 5–18 years with a restless sleep disorder were treated with FCM (*n* = 15) or ferrous sulphate (*n* = 15), all iron parameters tested were found to be significantly higher after FCM treatment compared with ferrous sulphate [26].

### Safety of FCM in children and adolescents

Safety findings in relation to the use of FCM in children and/or adolescents were reported in 25 of the 33 publications (Table 1) [21–23, 25–28, 30–41, 43, 45, 46, 50, 51, 53]. The reported incidence and types of adverse events (AEs) varied between the publications (Table 1) but were in line with those described in the prescribing information [17, 18].

Among the studies in children < 14 years old with IDA due to various causes, one retrospective study in 176 children reported hypotension (one patient), rash (four patients) and fever (two patients) [28]. In another retrospective study on 60 children, the authors identified three episodes associated with mild AEs (type of event not reported) and one episode of extravasation in a total of 65 episodes of FCM administration [23]. In a retrospective study in 51 children aged < 14 years with IDA of varying aetiologies [39], as well as in a retrospective study in 14 children aged < 2 years with intestinal failure and ID [21], the authors stated that no AEs were observed.

In the largest study to report on the safety of FCM across the paediatric age range (up to 18 years), which included 144 children with ID/IDA of varying aetiologies, 11 patients experienced in-hospital AEs and five patients reported AEs possibly related to FCM during the 96-h follow-up period after leaving the hospital [33]. In a study of 128 children with IBD and IDA, three patients reported an AE [22]. Twenty-five children had low serum phosphate, but only two children had severe hypophosphataemia requiring correction. There were no AEs in patients < 6 years old (*n* = 11). In another large study in children with IBD and ID/IDA, itch, urticarial rash and low-grade fever were reported in two of 101 patients [40]. In the studies that included FCM and other iron therapies, no notable differences were seen in the incidence of AEs between treatment groups [25, 26, 34, 41].

Two retrospective studies focused on the incidence of hypophosphataemia in children and adolescents following the administration of FCM for the treatment of IDA of various causes. The first included 36 children (22 females, 14 males; median age, 12.7 years) from a single centre, who had a total of 71 FCM infusions [35]. Hypophosphataemia occurred in six patients after the first dose and overall, after eight out of 71 infusions. Of the six patients with hypophosphataemia, five were female (three had IBD and two had errors of metabolism/mitochondrial disease). Multiple regression analysis detected gender-specific differences, with girls more likely to experience a decrease in plasma phosphate after the first dose. The authors also noted that the retrospective design of the study meant that systematic information on signs and symptoms of hypophosphatemia was lacking [35]. In a second study, in 225 subjects aged 2 months–20.3 years, hypophosphataemia occurred after 44 out of 313 FCM infusions, in 40 patients [30]. Of the 40 patients who developed hypophosphataemia, none had symptoms documented in the electronic health record, and seven were prescribed supplemental phosphate. It was found that a lower pre-infusion phosphate level was associated with the development of hypophosphataemia. In addition, a case series highlighted the occurrence of hypophosphataemia following FCM treatment in three adolescents with IBD [50].

## Discussion

The publications identified in this literature review indicate that FCM is an effective and generally well-tolerated treatment for ID or IDA of various aetiologies in children and adolescents. Although only three studies focused on children and adolescents under 14 years old [23, 28, 39], most of the other studies also included this age group (together with older children). There were no notable differences in the overall efficacy or safety findings in the studies in children < 14 years old as compared with the other studies in a wider age range.

The incidence of hypophosphataemia following FCM treatment in children [30, 35] appears to be lower than in adults [54, 55]. In one study, the authors also reported that girls were more likely to experience a decrease in plasma phosphate concentration after receiving FCM [35]; however, gender effects have not been observed in adults [54]. Although most clinical studies in adults report hypophosphataemia as “asymptomatic” or not associated with clinical sequelae [56], serum phosphate levels begin to recover approximately 2 weeks after FCM treatment [54, 55, 57]. Hypophosphataemia is an identified risk of FCM treatment that requires appropriate management, as elaborated in the prescribing information [17, 18]. The mechanism of hypophosphataemia following FCM administration is not well understood, but there is some evidence to suggest that it is caused by increased levels of intact fibroblast growth factor 23 (FGF23), leading to reduced serum phosphate [58, 59].

Only one small study was identified that compared FCM with another intravenous iron therapy, iron sucrose, in the paediatric IBD setting [41]. Statistical comparisons were not made between the two treatment groups, but similar efficacy results were observed. Iron sucrose is the most commonly used intravenous iron therapy [4] but is not approved for use in children in Europe [60]. In addition, iron sucrose treatment may involve repeated administration to achieve the desired dose [4]. In the aforementioned study, patients in the iron sucrose group received at least three administrations, and patients in the FCM group had a single administration [41].

This review highlights that most of the data currently available around the use of FCM in children or adolescents are from retrospective uncontrolled observational studies in single centres. Only two prospective studies were found [40, 41], but neither were randomised nor controlled. The studies had different patient inclusion criteria and endpoints, making them difficult to compare. Furthermore, only one study included long-term follow-up [41]. However, the literature search for this review was conducted using only two databases, PubMed and Embase. Another limitation is that a formal systematic review was not conducted; therefore, the risk of bias or certainty of evidence was not assessed.

To validate the efficacy of FCM in the paediatric population and to further investigate hypophosphataemia and other potential side effects, prospective, randomised controlled studies, with predefined endpoints, are urgently needed. Given that IV iron complexes are nanomedicines [19], each endowed with its unique therapeutic characteristics, the benefits of treatment with FCM in the paediatric setting, cannot be readily extrapolated to similar outcomes with other IV iron complexes. This is yet another legitimate reason mandating the need for robust clinical studies of FCM or any other IV iron complex to showcase an equivalent beneficial outcome in children, including toddlers and pre-schoolers, bearing in mind that FCM has also recently received FDA approval for > 1-year-olds. Recently, a randomised controlled study in 64 children with IBD aged 8–18 years (Prospective Open label study of Parenteral vs Enteral iron in Young IBD patients and Effect on physical fitness [POPEYE study]) was completed and found that FCM was superior to oral iron in terms of early improvement in physical fitness (based on 6-min walking distance) and that the increase in haemoglobin levels was similar for both groups [61]. There is also an ongoing randomised controlled study enrolling 76 children with IDA aged 1–17 years (ClinicalTrials.gov Identifier: NCT03523117); patients in this study whose response to the control preparation (oral iron) is unsatisfactory will be treated with FCM in a follow-on study (ClinicalTrials.gov Identifier: NCT04269707). The outcomes of the ongoing studies will help to build the evidence base for FCM in children and adolescents and have the potential to impact future clinical practice guidelines.

## Conclusions

The published evidence indicates that treatment with FCM is associated with improvements in iron status parameters and iron deficiency anaemia in children and adolescents, including those aged < 14 years old. FCM appears to be well tolerated in the paediatric setting, and potential risks of hypophosphataemia, if any, can be adequately managed in accordance with the prescribing information [17, 18]. The majority of the publications were retrospective studies, and it is known that a true causal relationship can be better established by well-designed prospective studies where there are options to minimise different types of bias. Therefore, it is now time to acknowledge ID and IDA as common conditions in paediatric populations and design prospective, randomised controlled studies, particularly in children with underlying conditions for which guidelines already recommend IV iron therapy, such as CKD [13], restless legs syndrome [15] and children on long-term parenteral nutrition [16]. Furthermore, well-designed prospective studies in children aged < 14 years will help to inform clinical and public health decisions on the use of FCM in this younger age group.

## Abbreviations

AE: Adverse event
CD: Crohn’s disease
CKD: Chronic kidney disease
ESR: Erythrocyte sedimentation rate
FCM: Ferric carboxymaltose
GI: Gastrointestinal
Ht: Haematocrit
Hb: Haemoglobin
IBD: Inflammatory bowel disease
ID: Iron deficiency
IDA: Iron deficiency anaemia
IRIDA: Iron-refractory iron deficiency anaemia
IV: Intravenous
MCH: Mean cell haemoglobin
MCV: Mean corpuscular volume
NBCD: Non-biological complex drug
TIBC: Total iron binding capacity
TSAT: Transferrin saturation

## References

[1] Safiri S, Kolahi AA, Noori M, Nejadghaderi SA, Karamzad N, Bragazzi NL, Sullman MJM, Abdollahi M, Collins GS, Kaufman JS, Grieger JA (2021). Burden of anemia and its underlying causes in 204 countries and territories, 1990–2019: results from the Global Burden of Disease Study 2019. J Hematol Oncol.

[2] Lopez A, Cacoub P, Macdougall IC, Peyrin-Biroulet L (2016). Iron deficiency anaemia. Lancet.

[3] Cappellini MD, Musallam KM, Taher AT (2020). Iron deficiency anaemia revisited. J Intern Med.

[4] Mantadakis E, Chatzimichael E, Zikidou P (2020). Iron deficiency anemia in children residing in high and low-income countries: risk factors, prevention, diagnosis and therapy. Mediterr J Hematol Infect Dis.

[5] GBD (2019). Risk Factors Collaborators (2020) Global burden of 87 risk factors in 204 countries and territories, 1990–2019: a systematic analysis for the Global Burden of Disease Study 2019. Lancet.

[6] van der Merwe LF, Eussen SR (2017). Iron status of young children in Europe. Am J Clin Nutr.

[7] Gupta PM, Perrine CG, Mei Z, Scanlon KS (2016). Iron, anemia, and iron deficiency anemia among young children in the United States. Nutrients.

[8] Christian P, Smith ER (2018). Adolescent undernutrition: global burden, physiology, and nutritional risks. Ann Nutr Metab.

[9] Mattiello V, Schmugge M, Hengartner H, von der Weid N, Renella R; SPOG Pediatric Hematology Working Group (2020). Diagnosis and management of iron deficiency in children with or without anemia: consensus recommendations of the SPOG Pediatric Hematology Working Group. Eur J Pediatr.

[10] Powers JM, Nagel M, Raphael JL, Mahoney DH, Buchanan GR, Thompson DI (2020). Barriers to and facilitators of iron therapy in children with iron deficiency anemia. J Pediatr.

[11] British Society of Paediatric Gastroenterology, Hepatology and Nutrition (2014) Diagnosis and management of anaemia in children with IBD Diagnosis and management of anaemia in children with IBD. Available at: http://mail.bspghan.org.uk/documents/Iron%20Deficiency%20PIBD%20Jan%202015-2. (Accessed Feb 2022)

[12] Goyal A, Zheng Y, Albenberg LG, Stoner NL, Hart L, Alkhouri R, Hampson K, Ali S, Cho-Dorado M, Goyal RK, Grossman A (2020). Anemia in children with inflammatory bowel disease: a position paper by the IBD Committee of the North American Society of Pediatric Gastroenterology, Hepatology and Nutrition. J Pediatr Gastroenterol Nutr.

[13] Kidney Disease: Improving Global Outcomes (KDIGO) Anemia Work Group. KDIGO Clinical Practice Guideline for Anemia in Chronic Kidney Disease (2012) Kidney Int Suppl 2:279–335.

[14] National Institute for Health and Care Excellence (NICE) (2021) Chronic kidney disease: assessment and management. Available at: https://www.nice.org.uk/guidance/ng203/resources/chronic-kidney-disease-assessment-and-management-pdf-66143713055173. (Accessed Feb 2022) 34672500

[15] Allen RP, Picchietti DL, Auerbach M, Cho YW, Connor JR, Earley CJ, Garcia-Borreguero D, Kotagal S, Manconi M, Ondo W, Ulfberg J, Winkelman JW; International Restless Legs Syndrome Study Group (IRLSSG) (2018) Evidence-based and consensus clinical practice guidelines for the iron treatment of restless legs syndrome/Willis-Ekbom disease in adults and children: an IRLSSG task force report. Sleep Med 41:27–44. 10.1016/j.sleep.2017.11.1126 10.1016/j.sleep.2017.11.1126 29425576

[16] Domellöf M, Szitanyi P, Simchowitz V, Franz A, Mimouni F (2018) ESPGHAN/ESPEN/ESPR/CSPEN working group on pediatric parenteral nutrition ESPGHAN/ESPEN/ESPR/CSPEN guidelines on pediatric parenteral nutrition: iron and trace minerals. Clin Nutr 37(6 Pt B):2354–2359. 10.1016/j.clnu.2018.06.949 10.1016/j.clnu.2018.06.949 30078716

[17] Ferinject (ferric carboxymaltose) summary of product characteristics (2021) Available at: https://www.medicines.org.uk/emc/product/5910/smpc. (Accessed Feb 2022)

[18] Injectafer prescribing information (2022) Available at: https://injectafer.com/. (Accessed May 2022)

[19] Gaspar RS, Silva-Lima B, Magro F, Alcobia A, da Costa FL, Feio J (2020). Non-biological complex drugs (NBCDs): complex pharmaceuticals in need of individual robust clinical assessment before any therapeutic equivalence decision. Front Med (Lausanne).

[20] Nikravesh N, Borchard G, Hofmann H, Philipp E, Flühmann B, Wick P (2020). Factors influencing safety and efficacy of intravenous iron-carbohydrate nanomedicines: from production to clinical practice. Nanomedicine.

[21] Athiana I, Waldenvik K, Paulsson M, Engstrand-Lilja H, Lundberg A, Finkel Y, Nyström N (2018). 5PSQ-017 safety of intravenous ferric carboxymaltose in treatment of iron deficiency in children under 2 years with intestinal failure. Eur J Hosp Pharm.

[22] Cococcioni L, Elzein A, Sider S, Chadokufa S, Buckingham R, Ocholi A, Shah N, McCartney S, Saliakellis E, Borrelli O, Kiparissi F (2019). P451 safety and efficacy of ferric carboxymaltose (FCM) for the treatment of iron deficiency anaemia in paediatric patients affected by inflammatory bowel disease (pIBD). J Crohns Colitis.

[23] Crighton G, Gurung A, Barnes C (2017). P-590 ferric carboxymaltose in children less than 14 years of age. Vox Sang.

[24] Dargan C, Simon D, Fleishman N, Goyal A, Sharma M (2020). A study examining the efficacy of ferric carboxymaltose in a large pediatric cohort. Blood.

[25] DelRosso LM, Ferri R, Allen RP, Chen ML, Kotagal S, Picchietti D (2020). 1001 intravenous ferric carboxymaltose for restless legs syndrome in children and adolescents. Sleep.

[26] DelRosso LM, Picchietti DL, Ferri R (2021) Comparison between oral ferrous sulfate and intravenous ferric carboxymaltose in children with restless sleep disorder. Sleep 44:zsaa155. 10.1093/sleep/zsaa155 10.1093/sleep/zsaa155 32840615

[27] Hachemi S, Mutalib M, Chadokufa S, Huggett B, Sider S, Shah N, Whitley L, McCartney S, Kiparissi F (2015). P377 assessment of safety and efficacy of ferric carboxymaltose (Ferinject®) in the management of iron deficiency with anaemia (IDA) or without anaemia in children and adolescents with inflammatory bowel disease (IBD). J Crohns Colitis.

[28] Hong MHS, Singh H, Hinds R (2019). Letter to the editor. J Paediatr Child Health.

[29] Jacobson-Kelly AE, Stanek JR, Powers JM, Dotson JL, O’Brien SH (2020). Trends in anemia, iron, therapy, and transfusion in hospitalized pediatric patients with inflammatory bowel disease. J Pediatr.

[30] Kirk SE, Scheurer ME, Bernhardt MB, Mahoney DH, Powers JM (2021). Phosphorus levels in children treated with intravenous ferric carboxymaltose. Am J Hematol.

[31] Knafelz D, Acton N, Angelakopoulou A, Sider S, Chadokufa S, Shah N, Kiparissi F (2017). P048 Intravenous ferric carboxymaltose is effective and safe for the treatment of iron deficiency anaemia in children affected by inflammatory bowel disease. Dig Liver Dis.

[32] Laass MW, Straub S, Chainey S, Virgin G, Cushway T (2014). Effectiveness and safety of ferric carboxymaltose treatment in children and adolescents with inflammatory bowel disease and other gastrointestinal diseases. BMC Gastroenterol.

[33] Ozsahin H, Schaeppi M, Bernimoulin M, Allard M, Guidard C, van den Ouweland F (2020). Intravenous ferric carboxymaltose for iron deficiency anemia or iron deficiency without anemia after poor response to oral iron treatment: benefits and risks in a cohort of 144 children and adolescents. Pediatr Blood Cancer.

[34] Papadopoulos M, Patel D, Korologou-Linden R, Goto E, Soondrum K, Fell JME, Epstein J (2018). Safety and efficacy of parenteral iron in children with inflammatory bowel disease. Br J Clin Pharmacol.

[35] Posod A, Schaefer B, Mueller T, Zoller H, Kiechl-Kohlendorfer U (2020). Hypophosphatemia in children treated with ferric carboxymaltose. Acta Paediatr.

[36] Powers JM, Shamoun M, McCavit TL, Adix L, Buchanan GR (2017). Intravenous ferric carboxymaltose in children with iron deficiency anemia who respond poorly to oral iron. J Pediatr.

[37] Sasankan N, Duncan H, Curtis L, McGuckin C, Shannon C, Barclay A, Fraser S, Nair M, Russell RK, Hansen R (2021). Ferric carboxymaltose across all ages in paediatric gastroenterology shows efficacy without increased safety concerns. J Pediatr Gastroenterol Nutr.

[38] Spinner JA, Puri K, Powers J, Dasari T, Tunuguntla H, Choudhry S, Cabrera AG, Shah M, Dreyer WJ, Denfield SW, Price JF (2019). Abstract 13788: intravenous iron replacement therapy with ferric carboxymaltose is safe and effective in pediatric patients with heart failure. Circulation.

[39] Tan MLN, Windscheif PM, Thornton G, Gaynor E, Rodrigues A, Howarth L (2017). Retrospective review of effectiveness and safety of intravenous ferric carboxymaltose given to children with iron deficiency anaemia in one UK tertiary centre. Eur J Pediatr.

[40] Carman N, Muir R, Lewindon P (2019) Ferric carboxymaltose in the treatment of iron deficiency in pediatric inflammatory bowel disease. Transl Pediatr 8:28–34. 10.21037/tp.2019.01.01 10.21037/tp.2019.01.01 PMC6382504 30881896

[41] Valério de Azevedo S, Maltez C, Lopes AI (2017). Pediatric Crohn’s disease, iron deficiency anemia and intravenous iron treatment: a follow-up study. Scand J Gastroenterol.

[42] Abdelmahmuod EA, Yassin MA (2020). Iron deficiency anemia-induced lymphocytopenia in a young female. Case Rep Oncol.

[43] Beverina I, Macellaro P, Parola L, Brando B (2018). Extreme anemia (Hb 33 g/L) in a 13-year-old girl: is the transfusion always mandatory?. Transfus Apher Sci.

[44] Daignault C, Gulati N, Olive A, Ruan W, Fishman D, Narine K (2020). Poster #758 Gastric Burkitt lymphoma diagnosed after work up for severe iron deficiency anemia: a case report. Pediatr Blood Cancer.

[45] Harris RE, Garrick V, Curtis L, Russell RK (2020). Skin staining due to intravenous iron extravasation in a teenager with Crohn’s disease. Arch Dis Child.

[46] Hönemann CW, Doll D, Kampmeier T, Ertmer C, Hagemann O, Hahnenkamp K (2012). Anaemia tolerance: bridging with intravenous ferric carboxymaltose in a patient with acute post-haemorrhagic anaemia. Br J Anaesth.

[47] Joseph M, Szafron V, Yang B, Srivaths L, Anvari S, Castells M, Noroski L (2019). M030 Ferric carboxymaltose desensitization in refractory idiopathic iron-deficiency anemia, iron-infusion anaphylaxis, severe atopy and hypertryptasemia. Ann Allergy Asthma Immunol.

[48] Pérez-Ferrer A, Gredilla E, de Vicente J, Laporta Y (2012). Cardiac surgery without blood products in a Jehovah’s Witness child with factor VII deficiency. J Cardiothorac Vasc Anesth.

[49] Shrinkhal, Singh A, Agrawal A, Yadav P, Verma R (2020) Sudden vision loss as first clinical manifestation of anaemic retinopathy. J Clin Diag Res 14:ND01–ND03. 10.7860/JCDR/2020/44332.13706

[50] Harris RE, Armstrong L, Curtis L, Garrick V, Gervais L, Tayler R, Hansen R, Russell RK (2019). Severe hypophosphataemia following ferric carboxymaltose infusion in paediatric patients with inflammatory bowel disease. Frontline Gastroenterol.

[51] Crook K, Tyrrell T, Hyer W (2018). N035 The use of intravenous (IV) ferric carboxymaltose in a paediatric IBD population. J Crohns Colitis.

[52] Jones J, Butcher A, Rodgers T, Farrell C, Blackman N (2018). Poster number: 007 Pharmacokinetic/pharmacodynamic modeling of intravenous ferric carboxymaltose in pediatric subjects with iron deficiency anemia. Clin Pharmacol Drug Dev.

[53] Mantadakis E, Roganovic J (2017). Safety and efficacy of ferric carboxymaltose in children and adolescents with iron deficiency anemia. J Pediatr.

[54] Wolf M, Chertow GM, Macdougall IC, Kaper R, Krop J, Strauss W (2018). Randomized trial of intravenous iron-induced hypophosphatemia. JCI Insight.

[55] Wolf M, Rubin J, Achebe M, Econs MJ, Peacock M, Imel EA, Thomsen LL, Carpenter TO, Weber T, Brandenburg V, Zoller H (2020). Effects of iron isomaltoside vs ferric carboxymaltose on hypophosphatemia in iron-deficiency anemia: two randomized clinical trials. JAMA.

[56] Glaspy JA, Lim-Watson MZ, Libre MA, Karkare SS, Hadker N, Bajic-Lucas A, Strauss WE, Dahl NV (2020). Hypophosphatemia associated with intravenous iron therapies for iron deficiency anemia: a systematic literature review. Ther Clin Risk Manag.

[57] Hussain I, Bhoyroo J, Butcher A, Koch TA, He A, Bregman DB (2013) Direct comparison of the safety and efficacy of ferric carboxymaltose versus iron dextran in patients with iron deficiency anemia. Anemia 2013:169107. 10.1155/2013/169107 10.1155/2013/169107 PMC3773415 24069536

[58] Wolf M, Koch TA, Bregman DB (2013) Effects of iron deficiency anemia and its treatment on fibroblast growth factor 23 and phosphate homeostasis in women. J Bone Miner Res 28:1793–1803. 10.1002/jbmr.1923 10.1002/jbmr.1923 23505057

[59] Huang LL, Lee D, Troster SM, Kent AB, Roberts MA, Macdougall IC, McMahon LP (2018) A controlled study of the effects of ferric carboxymaltose on bone and haematinic biomarkers in chronic kidney disease and pregnancy. Nephrol Dial Transplant 33:1628–1635. 10.1093/ndt/gfx310 10.1093/ndt/gfx310 29165637

[60] Venofer (iron sucrose) Summary of product characteristics (2021) Available at: https://www.medicines.org.uk/emc/product/5911/smpc#gref. (Accessed May 2022)

[61] Bevers N, van de Vijver E, Aliu A, Rezazadeh Ardabili A, Rosias P, Busari J, Stapelbroek J, Bertrams I, van der Feen C, Oudshoorn A, Teklenburg S, Escher J, Vande Velde S, Winkens B, Raijmakers M, Vreugdenhil A, Pierik M, van Rheenen P (2021). P398 effect of intravenous versus oral iron therapy on physical fitness and haemoglobin in paediatric IBD patients with anaemia. J Crohns Colitis.

